# An Examination of the Association between FOXA1 Staining Level and Biochemical Recurrence following Salvage Radiation Therapy for Recurrent Prostate Cancer

**DOI:** 10.1371/journal.pone.0151785

**Published:** 2016-03-17

**Authors:** Michael G. Heckman, Jessica L. Robinson, Katherine S. Tzou, Alexander S. Parker, Kevin J. Wu, Tracy W. Hilton, William J. Howat, Jodi L. Miller, Pamela A. Kreinest, Thomas M. Pisansky, Steven E. Schild, Jennifer L. Peterson, Laura A. Vallow, Jason S. Carroll, Steven J. Buskirk

**Affiliations:** 1 Department of Health Sciences Research, Mayo Clinic, Jacksonville, Florida, United States of America; 2 Cancer Research UK Cambridge Institute, University of Cambridge, Cambridge, United Kingdom; 3 Department of Radiation Oncology, Mayo Clinic, Jacksonville, Florida, United States of America; 4 Department of Pathology, Mayo Clinic, Jacksonville, Florida, United States of America; 5 Department of Cancer Biology, Mayo Clinic, Jacksonville, Florida, United States of America; 6 Department of Radiation Oncology, Mayo Clinic, Rochester, Minnesota, United States of America; 7 Department of Radiation Oncology, Mayo Clinic, Scottsdale, Arizona, United States of America; University of Kentucky College of Medicine, UNITED STATES

## Abstract

**Background:**

Standardly collected clinical and pathological patient information has demonstrated only moderate ability to predict risk of biochemical recurrence (BCR) of prostate cancer in men undergoing salvage radiation therapy (SRT) for a rising PSA after radical prostatectomy (RP). Although elevated FOXA1 staining has been associated with poor patient outcomes following RP, it has not been studied in the specific setting of SRT after RP. The aim of this study was to evaluate the association between FOXA1 staining level and BCR after SRT for recurrent prostate cancer.

**Methods:**

A total of 141 men who underwent SRT at our institution were included. FOXA1 staining levels in primary tumor samples were detected using immunohistochemistry. FOXA1 staining percentage and intensity were measured and multiplied together to obtain a FOXA1 H-score (range 0–12) which was our primary staining measure. P-values ≤ 0.0056 were considered as statistically significant after applying a Bonferroni correction for multiple comparisons.

**Results:**

There was not a significant association between FOXA1 H-score and risk of BCR when considering H-score as an ordinal variable or as a categorical variable (all P≥0.090). Similarly, no significant associations with BCR were observed for FOXA1 staining percentage or staining intensity (all P≥0.14).

**Conclusions:**

FOXA1 staining level does not appear to have a major impact on risk of BCR after SRT.

## Introduction

Each year in the United States approximately 75,000 men will undergo a radical prostatectomy (RP) for localized prostate cancer [1]. Of these, between 15% and 25% will experience a significant rise in their serum prostate specific antigen level following surgery, indicating a biochemical recurrence (BCR) of their disease [1]. The only potentially curative treatment option available to men who have experienced BCR of prostate cancer after RP is salvage radiation therapy (SRT); however, the reported success of SRT have ranged from 10% to 65% [2–10]. As such, the ability to accurately identify which men have the highest likelihood of responding to SRT represents a key clinical issue for the field. Indeed, the ability to accurately forecast which men are likely to respond to SRT is important in order to optimize the selection of patients for this treatment, and to design better clinical trials to evaluate novel means of improving SRT efficacy. Related to this, a number of studies have been performed that attempt to identify specific characteristics that are associated with risk of BCR after SRT. Several well-replicated risk factors have been established, such as elevated pre-SRT PSA level, more advanced pathological tumor stage, higher Gleason score, negative surgical margin, and shorter pre-SRT PSA doubling time [2–10].

In an attempt to provide patients with individualized estimates of BCR risk that are tailored to their clinical and pathological characteristics, several large studies have proposed scoring algorithms that combine information from multiple prognostic factors [4–5, 10]. However, the ability of these algorithms to stratify risk of BCR has considerable room for improvement; for example, a notable proportion of patients in the low BCR risks groups still experience BCR [4–5, 8–10]. Therefore, there is a need to identify other factors that are predictive of BCR after SRT in order to enable better patient selection for SRT. Given that the aforementioned scoring algorithms have incorporated only standard clinical and pathological information that is available to physicians at the time of SRT, the study of tumor-based biomarkers may be useful in this regard.

The forkhead transcription factor FOXA1 is emerging as a critical player in prostate cancer biology. Often described as a pioneer factor, FOXA1 has the ability to bind to highly compacted chromatin and make these regions more accessible to other transcription factors such as the androgen receptor (AR) [11]. In the human adult prostate, FOXA1 is expressed in the epithelial cells of the peripheral zone [12], the site from which the majority of prostate cancers originate [13]. In prostate cancer cell lines, FOXA1 is required for androgen receptor activation [14–16], and over-expression of FOXA1 increases AR binding throughout the genome [17]. Results from clinical studies have indicated that high levels of FOXA1 have been associated with an increased risk of BCR [12, 17–18] and prostate cancer-specific death [15] after RP. In the context of recurrent disease, FOXA1 has been shown to be expressed in 90% of prostate cancer metastases [19], and a number of studies determined that the genes located adjacent to FOXA1 binding sites in cell line models of castrate resistance prostate cancer are strongly associated with gene signatures of prostate cancer recurrence [17, 20]. However, the ability of FOXA1 expression to predict risk of BCR in the specific patient population of men undergoing SRT for recurrent prostate cancer after RP has not been assessed to date. The aforementioned reports of associations between high FOXA1 staining levels and poor patient outcomes, combined with the fact that FOXA1 expression appears to persist in metastases, suggest that FOXA1 is a marker of disease aggressiveness and is a reasonable candidate for study in relation to BCR in SRT patients. Therefore, in this study we evaluated the association of immunohistochemical staining levels of FOXA1 in primary prostate cancer tumor samples with risk of BCR after SRT.

## Materials and Methods

### Patient Selection and Outcome Definition

All 141 patients who underwent SRT to treat a rising PSA following RP for prostate cancer at the Mayo Clinic between July 1987 and February 2003 and who had archived tumor tissue available were included in this study. Information was retrospectively collected from patients’ charts regarding baseline clinical information (pre-RP PSA, pre-SRT PSA, age, pre-SRT hormone therapy), pathological information (pathological tumor stage, surgical margin, Gleason score), SRT information (SRT dose, length of time between RP to SRT initiation), and post-SRT PSA measurements. BCR was the primary endpoint of this study and was defined as the occurrence of a PSA value of 0.2 ng/ml or greater and rising following the post-SRT nadir. The date of BCR was considered to be the date of the defining PSA value without backdating. The Mayo Clinic Institutional Review Board approved the study and all subjects provided written informed consent.

### Salvage radiation therapy information

Patients were immobilized in the supine position with contrast in the bladder and rectum, and the majority also had a retrograde urethrography performed. The prostatic fossa was treated with 6–20 MV photons to a median dose of 64.8 Gy (Range: 58.4–72.4 Gy) in daily 1.8–2.0 Gy fractions. Computed tomography-based treatment planning was utilized in most cases. Custom blocking and 4–8 stationary conformal or rotational fields were used. Intensity modulation, inverse planning, and image-guidance techniques were not available for use at our institution during this timeframe. After completion of treatment, patients were typically evaluated every 3–4 months for 2 years, and every 6–12 months thereafter.

### Immunohistochemistry

An experienced uropathologist selected the tumor block from the original prostatectomy specimen with the highest-grade tumor. Five-μm-thick slides were cut and stained by immunohistochemical (IHC) methods for FOXA1. All IHC staining was performed on the fully automated Leica Bond III (Leica Microsystems, Milton Keynes, UK) using the Polymer Refine kit with DAB Enhancer (Leica). Briefly, following antigen retrieval, primary antibody was applied for 15 minutes at room temperature, followed by anti-rabbit polymer-HRP for 8 minutes, Diaminobenzidine (DAB) for 10 minutes and DAB enhancer for a further 10 minutes. All steps were completed at room temperature and wash steps in Leica Bondwash were included between each step. Leica Haematoxylin was applied for 5 minutes for nuclear counterstaining. Rabbit anti-FOXA1 (Abcam, UK; ab23738) was validated previously on human breast tissue and was used for the study at 1:800 and diluted in antibody diluent consisting of 1% Donkey Serum, 0.05% Tween20 in 300mM TBS to reduce background staining. Antigen retrieval was performed using Leica ER1 (Sodium Citrate pH 6) for 20 mins at 100°C. The staining was conducted in two batches, using anti-FOXA1 lot numbers GR77830-1 for the first batch (N = 73) and GR130594-1 for the second batch (N = 68). A lot comparison was performed prior to staining and it was determined that the staining was equivalent

**Fig 1:**
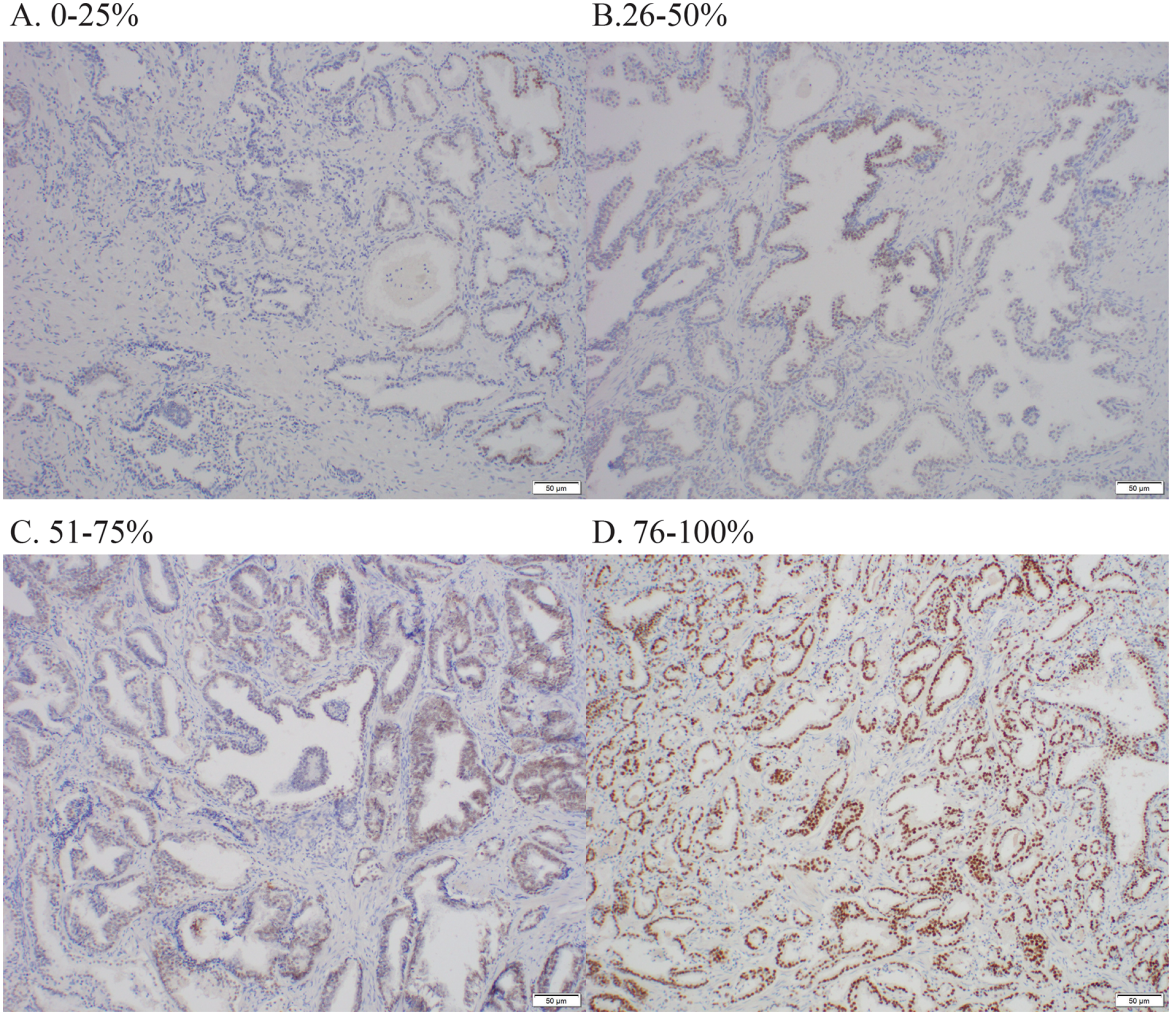
Representative immunostaining showing examples of prostate tumors with (A) 0–25% staining percentage, (B) 26–50% staining percentage, (C) 51–75% staining percentage, and (D) 76–100% staining percentage.

**Fig 2:**
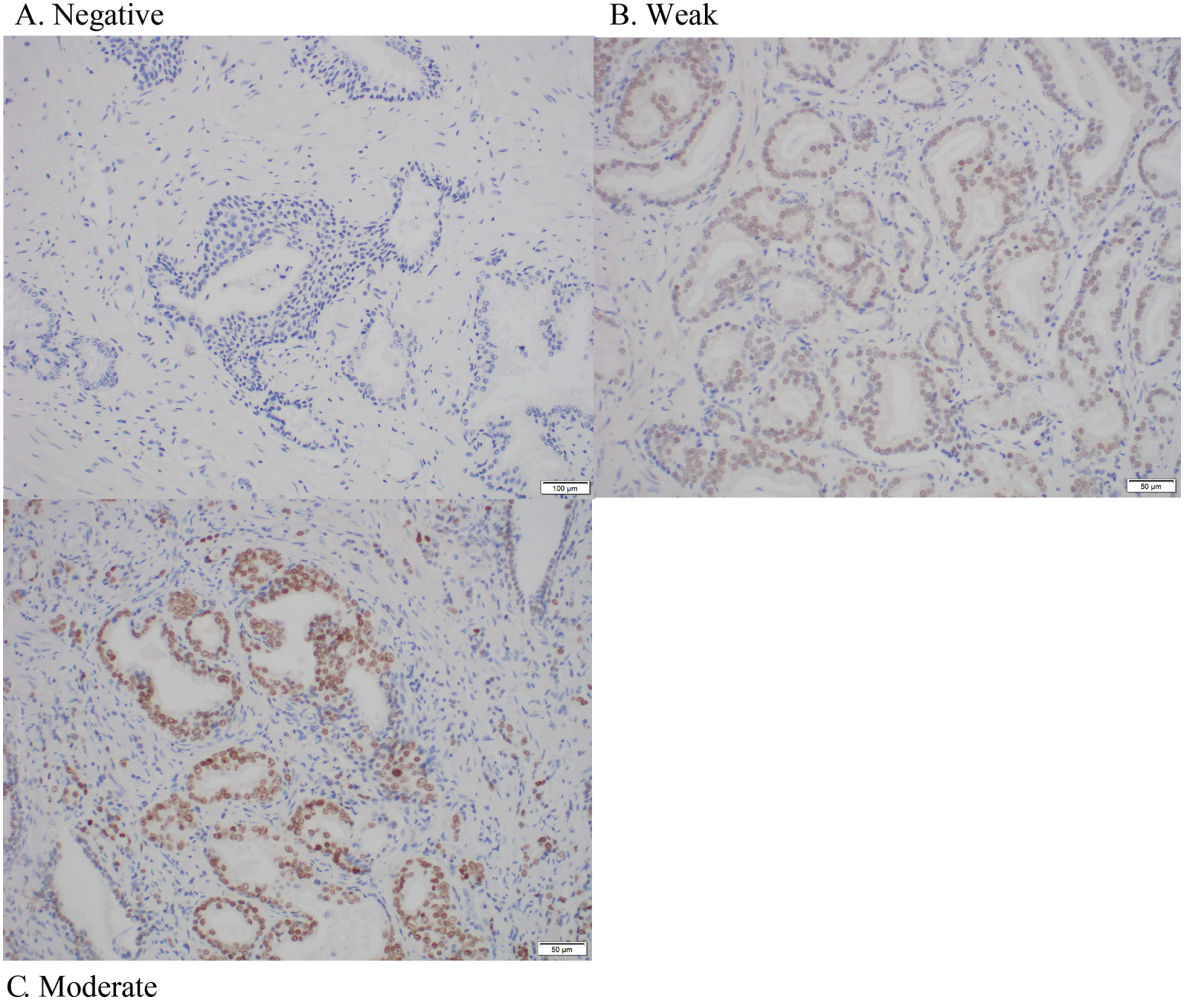
Representative immunostaining showing examples of prostate tumors with (A) negative staining intensity, (B) weak staining intensity, and (C) moderate staining intensity.

After IHC staining was completed, the percentage of cells with nuclear staining (i.e. FOXA1 staining percentage) as well as staining intensity were measured manually by an experienced uropathologist who was blinded to patient characteristics and outcomes. FOXA1 staining percentage was scored as 1 of 4 categories (1 = 0–25%, 2 = 26–50%, 3 = 51–75%, 4 = 76–100%), as was FOXA1 staining intensity (0 = negative, 1 = weak, 2 = moderate, 3 = strong). FOXA1 H-score was calculated by multiplying staining percentage and staining intensity, resulting in an H-score that has possible values ranging from 0 to 12. FOXA1 H-score was considered to be our primary FOXA1 staining measure since this semi-quantitative measure is most informative by taking into account both total protein levels and overall tumor burden [21]. FOXA1 staining percentage and staining intensity were considered as secondary measures. Examples of FOXA1 immunostaining for the patients in our study are provided in Fig 1 (staining percentage) and Fig 2 (staining intensity).

### Statistical Analysis

Continuous variables were summarized using the sample median and range. Patient characteristics were compared according to FOXA1 H-score when dichotomizing by the median value (H-score ≤ 3 vs. H-score >3) using Fisher’s exact test or a Wilcoxon rank sum test. The Kaplan-Meier method was used to estimate the cumulative incidence of BCR after SRT, censoring at the date of last PSA measurement for patients who did not experience BCR. Associations between patient characteristics and BCR after SRT were examined using unadjusted Cox proportional hazards regression models. Relative risks (RRs) and 95% confidence intervals (CIs) were estimated. Due to their skewed distributions, pre-RP PSA, pre-SRT PSA, and length of time from RP to SRT initiation were considered on the logarithm score in all Cox regression analysis.

Associations of each of the three different FOXA1 staining measures (H-score [primary measure], staining percentage, staining intensity) with BCR after SRT were evaluated using single variable and multivariable Cox proportional hazards regression models. Single variable models were adjusted for staining batch only in order to account for any small batch-to-batch variability in FOXA1 staining levels. In order to account for other potential confounding variables, multivariable models were adjusted for staining batch as well as any characteristic that either differed according to FOXA1 H-score (≤3 vs. >3) with a p-value ≤ 0.10, or displayed an association with BCR with a p-value of ≤ 0.10. Three different cutpoints were considered for both staining percentage (≤25% vs. >25%, ≤50% vs. >50%, ≤75% vs. >75%) and H-score (≤2 vs. >2, ≤3 vs. >3, ≤4 vs. >4) for use in Cox regression analysis. Cutpoints for staining percentage were chosen at each different level of this categorical variable, and cutpoints for H-score were chosen based on the sample 25^th^, 50^th^, and 75^th^ percentiles. For staining intensity, due to the small number of patients with negative staining, we dichotomized this variable as negative/weak vs. moderate in all association analysis. FOXA1 H-score and staining percentage were also examined as ordinal variables in Cox regression analysis; RRs in this ordinal variable analysis correspond to a 1-unit increase in H-score, and to a score increase of 1 for staining percentage (i.e. a change from 0–25% to 26–50%). We utilized a Bonferroni adjustment for multiple comparisons, after which p-values ≤ 0.0056 were considered as statistically significant. All statistical analysis was performed using R Statistical Software (version 2.14.0; R Foundation for Statistical Computing, Vienna, Austria).

## Results

A summary of FOXA1 staining levels for the cohort of 141 SRT patients is provided in Table 1. FOXA1 staining percentages were observed across the entire 0% to 100% spectrum, while FOXA1 staining intensity was most commonly either weak (71.6%) or moderate (24.1%), with no strong FOXA1 staining intensity observed for any patient. FOXA1 H-score, which as previously mentioned was our primary measure and was calculated by multiplying FOXA1 staining percentage and intensity, was between 0 and 2 for 56 patients (39.7%), between 3 and 4 for 60 patients (42.6%), and greater than 4 for 25 patients (17.7%).

**Table 1:** Summary of FOXA1 staining.

FOXA1 staining measure	No. (%) of patients (N = 141)
Staining percentage	
0%–25%	37 (26.2%)
26%–50%	22 (15.6%)
51%–75%	26 (18.4%)
76%–100%	56 (39.7%)
Staining intensity	
Negative	6 (4.3%)
Weak	101 (71.6%)
Moderate	34 (24.1%)
Strong	0 (0.0%)
H-score	
0	6 (4.3%)
1	25 (17.7%)
2	25 (17.7%)
3	21 (14.9%)
4	39 (27.7%)
6	5 (3.5%)
8	20 (14.2%)

FOXA1 H-score was calculated by multiplying FOXA1 staining percentage (1 = 0–25%, 2 = 26–50%, 3 = 51–75%, 4 = 76–100%) and FOXA1 staining intensity (0 = negative, 1 = weak, 2 = moderate, 3 = strong) resulting in an H-score that has possible values ranging from 0 to 12.

Table 2 displays a comparison of characteristics according to FOXA1 H-score when dichotomizing H-score according to the sample median (i.e. ≤3 vs. >3). Compared to patients with a lower FOXA1 H-score, there was a shorter duration between RP and SRT initiation (Median: 11.7 vs. 15.5 months, P = 0.061) and also a tendency to more often be in the first staining batch (67.2% vs. 39.0, P = 0.001) for patients with a higher FOXA1 H-score. There were no other notable differences in characteristics between these two FOXA1 staining groups (all P≥0.14, Table 2).

**Table 2:** Comparison of patient characteristics according to FOXA1 H-score.

Variable	FOXA1 H-Score 0–3 (N = 77)	FOXA1 H-Score 4–8 (N = 64)	P-value
Pre-RP PSA (ng/mL)	8.8 (1.6, 219.0)	10.5 (2.0, 155.0)	0.25
Pre-SRT PSA (ng/mL)	0.6 (0.1, 4.9)	0.5 (0.1, 15.3)	0.14
SRT dose (Gy)	64.8 (58.4, 70.2)	65.0 (59.4, 72.4)	0.27
Age	67.0 (46.1, 81.1)	66.2 (44.0, 80.7)	0.49
Length of time from RP to SRT initiation (months)	15.5 (3.0, 71.9)	11.7 (1.5, 99.3)	0.061
Pathological tumor stage			0.45
T2	14 (18.2%)	7 (10.9%)	
T3a	40 (51.9%)	39 (60.9%)	
T3b	23 (29.9%)	18 (28.1%)	
Surgical margin			0.61
Positive	44 (57.1%)	40 (62.5%)	
Negative	33 (42.9%)	24 (37.5%)	
Gleason score			0.48
3–6	27 (35.1%)	28 (45.2%)	
7	32 (41.6%)	23 (37.1%)	
8–10	18 (23.4%)	11 (17.7%)	
Pre-SRT hormone therapy			0.66
Yes	13 (16.9%)	13 (20.3%)	
No	67 (83.1%)	51 (79.7%)	
Staining batch			0.001
First	30 (39.0%)	43 (67.2%)	
Second	47 (61.0%)	21 (32.8%)	

The sample median (minimum, maximum) is given for continuous variables. P-values result from a Kruskal-Wallis rank sum test (continuous variables) or Fisher’s exact test (categorical variables). Information was unavailable regarding pre-RP PSA (N = 5) and Gleason score (N = 2). RP = radical prostatectomy; PSA = prostate-specific antigen; SRT = salvage radiation therapy.

With a median follow-up length of 8.8 years (Range: 0.4–22.2 years), 90 patients (63.8%) experienced BCR after SRT. Of the 51 patients who did not experience BCR, 15 died at a median of 2.1 years (Range: 0.1–7.5 years) after their last PSA measurement. At 5 and 10 years after the start of SRT, cumulative incidences of BCR were 53.9% (95% CI: 44.7%–61.6%) and 65.6% (95% CI: 55.8%–73.2%), respectively. In order to better understand how patient characteristics may act as confounders when examining the association between FOXA1 staining level and BCR in our primary analysis, associations between these characteristics and BCR are shown in S1 Table. Briefly, in our cohort of 141 patients, risk of BCR after SRT was significantly higher for patients with higher pre-SRT PSA levels (P<0.001), patients with a more advanced pathological tumor stage (P = 0.003), and patients with a higher Gleason score (P = 0.042).

In evaluation of the primary aim of the study, associations between each of the three FOXA1 staining measures (H-score [primary measure], staining percentage, staining intensity) and BCR after SRT are displayed in Table 3. In single variable analysis adjusting only for staining batch, there was not a significant association between BCR and FOXA1 H-score as either an ordinal variable (RR: 1.05, P = 0.33) or as a categorical variable for any of the cutpoints that were considered (all P≥0.090, Table 3). This lack of a significant association was consistent in multivariable analysis additionally adjusting for the potential confounding influences of length of time between RP and SRT, pre-SRT PSA, pathological tumor stage, and Gleason score (Table 3). When evaluating FOXA1 staining percentage and staining intensity individually, there were also no statistically significant associations with risk of BCR in single variable analysis or multivariable analysis (all P≥0.14, Table 3).

**Table 3:** Association between FOXA1 staining level and biochemical recurrence following salvage radiation therapy.

			Single variable analysis		Multivariable analysis	
FOXA1 staining measure & group	N	Cumulative 5-year incidence of BCR, % (95% CI)	RR (95% CI)	P-value	RR (95% CI)	P-value
FOXA1 H-score						
Ordinal variable	141	N/A	1.05 (0.95, 1.16)	0.33	1.06 (0.96, 1.18)	0.25
≤2	56	56.8 (41.1, 68.3)	1.00 (reference)	N/A	1.00 (reference)	N/A
>2	85	52.1 (39.9, 61.8)	0.91 (0.60, 1.39)	0.67	0.98 (0.63, 1.51)	0.93
≤3	77	58.3 (45.4, 68.2)	1.00 (reference)	N/A	1.00 (reference)	N/A
>3	64	48.7 (34.5, 59.9)	0.85 (0.55, 1.31)	0.45	1.06 (0.67, 1.69)	0.80
≤4	116	52.2 (41.9, 60.6)	1.00 (reference)	N/A	1.00 (reference)	N/A
>4	25	62.2 (36.8, 77.4)	1.57 (0.93, 2.63)	0.090	1.61 (0.93, 2.82)	0.092
FOXA1 staining percentage						
Ordinal variable	141	N/A	1.02 (0.86, 1.20)	0.85	1.09 (0.91, 1.31)	0.33
≤25%	37	50.6 (30.6, 64.8)	1.00 (reference)	N/A	1.00 (reference)	N/A
>25%	104	55.3 (44.4, 64.1)	1.09 (0.67, 1.75)	0.74	1.05 (0.64, 1.73)	0.84
≤50%	59	53.7 (38.7, 65.1)	1.00 (reference)	N/A	1.00 (reference)	N/A
>50%	82	54.1 (41.6, 63.9)	1.02 (0.67, 1.56)	0.92	1.19 (0.78, 1.83)	0.42
≤75%	85	55.1 (42.9, 64.6)	1.00 (reference)	N/A	1.00 (reference)	N/A
>75%	56	52.2 (36.7, 64.0)	1.02 (0.66, 1.57)	0.94	1.42 (0.89, 2.27)	0.14
FOXA1 staining intensity						
Negative or weak	107	55.8 (45.0, 64.5)	1.00 (reference)	N/A	1.00 (reference)	N/A
Moderate	34	48.3 (28.1, 62.8)	1.03 (0.63, 1.68)	0.92	0.84 (0.50, 1.41)	0.51

RRs, 95% CIs, and p-values result from Cox proportional hazards regression models. Single variable models were adjusted for staining batch (first or second). In addition to staining batch, multivariable models were also adjusted for any variable that differed between patients with a low and high FOXA1 H-score (i.e. H-score 1–6 vs. 7–12) with a p-value of 0.10 or lower, or any variable that was associated with BCR with a p-value of 0.10 or lower. Therefore, the variables adjusted for in the multivariable models were staining batch, time from RRP to SRT, pre-SRT PSA, pathological tumor stage, and Gleason score. Three separate dichotomizations of H-score were examined in Cox regression analysis that were defined based on the 25^th^ percentile (H-score = 2), the 50^th^ percentile (H-score = 3), and the 75^th^ percentile (H-score = 4). FOXA1 staining percentage was scored as one of four categories (0%–25%, 26%–50%, 51%–75%, 76%–100%) and therefore the three possible dichotomizations of this variable were examined in Cox regression analysis. Given that there were only 6 patients with negative FOXA1 staining intensity, we combined this group with the 101 patients who had weak FOXA1 staining intensity for comparison with patients who had moderate FOXA1 intensity staining in Cox regression analysis. BCR = biochemical recurrence; RR = relative risk; CI = confidence interval.

We performed several additional analyses related to staining batch in order to further address any potential influence it could have on our results. First, we examined the association between each FOXA1 staining measure and risk of BCR separately in the first and second staining batches. As shown in S2 Table, the lack of association between FOXA1 staining level and BCR was consistent in the two separate batches. Second, we removed all adjustments for staining batch from our Cox proportional hazards regression analyses involving the overall patient cohort. Similar results were observed, with no statistically significant associations between FOXA1 staining level and BCR risk (S3 Table).

The raw data utilized to perform the analysis is provided in S1 File.

## Discussion

This is the first study to date that assesses the ability of FOXA1 staining levels in primary prostate cancer tumors to predict BCR among men undergoing SRT. Based on our results, FOXA1 staining level does not appear to have a major impact on risk of BCR after SRT, as evidenced by the lack of a significant association with BCR for any of the FOXA1 staining variables that were examined. This lack of association remained apparent even after adjustment for key clinical and pathological factors known to be associated with risk of BCR after SRT and is especially apparent when considering that p-value of 0.0056 or lower were considered as statistically significant in order to account for multiple testing. Overall it seems relatively unlikely that FOXA1 staining level alone will be useful in improving risk stratification of BCR following SRT. Therefore, given the aforementioned need to identify new risk factors for BCR in order to optimize patient selection for SRT, it appears that biomarkers other than FOXA1 should be considered for study.

Although a major association between FOXA1 staining level and risk of BCR after SRT was not observed in our study, relatively strong associations have been noted by several groups in the context of BCR after RP. In a study of 102 RP patients from the United Kingdom, Robinson et al. observed a 5-fold increased risk of BCR in individuals with higher FOXA1 staining (P = 0.028) [17]. In a smaller study of 52 Japanese RP patients by Imamura et al, freedom from BCR was 88% in patients with low FOXA1 staining compared to 50% in patients with high staining (P = 0.011) [18]. Gerhardt et al. evaluated 207 patients from who had undergone RP in Switzerland and observed a mean time to BCR of 70 months in patients with high FOXA1 staining levels compared to a mean of 87 months in patients with low staining (P = 0.018) [12]. The fact that the degree of association between FOXA1 staining and BCR was not as strong in our study may be due the lower degree of variability in disease aggressiveness in our patient subgroup that all have recurrent and therefore more aggressive cancer. There also appears to be conflicting evidence regarding the specific role that FOXA1 plays in the context of recurrent disease. While numerous studies have emphasized the importance of FOXA1 in primary prostate cancer growth [22], its role in metastatic disease is yet to be fully defined. FOXA1 binding sites are located near key genes involved in castrate resistant prostate cancer [18, 20]. However, there is conflicting evidence regarding its role in cell motility and epithelial-to-mesenchymal transition, and FOXA1 may even inhibit metastasis formation in mice models [12, 23]. Overall, the evidence to date suggests that FOXA1 may be a useful biomarker for predicting initial disease progression, but not necessarily of progression after the treatment of recurrent disease by SRT.

Several limitations of our study are important to bear in mind. The retrospective design potentially introduces bias into the data collection. Additionally, the sample size of 141 patients is relatively small, and this results in a lack of power to detect associations between FOXA1 staining level and BCR. Therefore, the possibility of type II error (i.e. a false-negative finding) is also important to consider, and 95% confidence limits should be emphasized when interpreting our results as these may contain clinically meaningful effect sizes. Also, we observed that patients with a higher FOXA1 H-score tended to be more often in the first staining batch. All possible strategies to minimize batch to batch variation were employed, and therefore it is highly likely that this difference is due to a true difference in FOXA1 staining levels between the two batches. Indeed, when analyzing each batch separately, a similar lack of association between FOXA1 staining level and BCR was observed. Finally, the patient population of our tertiary referral center consists mostly of Caucasians (>98%), and therefore we cannot extend our findings to individuals of other races.

## Conclusions

The results of our study provide evidence that FOXA1 staining level alone is not dramatically associated with risk of BCR after SRT and is therefore relatively unlikely to be of use in improving patient selection for SRT following recurrent prostate cancer after RP. It appears that it may be more fruitful to consider other biomarkers for study in order to further optimize risk stratification for SRT candidates.

## Supporting Information

S1 File Raw data. (XLSX)

S1 Table Associations between patient characteristics and biochemical recurrence following salvage radiation therapy. (DOC)

S2 Table Associations between FOXA1 staining level and biochemical recurrence following salvage radiation therapy separately for patients in the first and second staining batches. (DOC)

S3 Table Associations between FOXA1 staining level and biochemical recurrence following salvage radiation therapy without adjusting for staining batch. (DOC)
