# Supplementary material for: An Examination of the Association between FOXA1 Staining Level and Biochemical Recurrence following Salvage Radiation Therapy for Recurrent Prostate Cancer
Source: PLoS One. 2016 Mar 17;11(3):e0151785. doi: 10.1371/journal.pone.0151785 (PMC4795739; doi:10.1371/journal.pone.0151785)
Supplement: S1 Table — (DOC) [file pone.0151785.s002.doc]

**S1 Table: Associations between patient characteristics and biochemical recurrence following salvage radiation therapy**

| Variable | RR (95% CI) | P-value |
| --- | --- | --- |
| Pre-RP PSA (doubling) | 1.09 (0.92, 1.29) | 0.31 |
| Pre-SRT PSA (doubling) | 1.38 (1.16, 1.65) | <0.001 |
| SRT dose (5 Gy increase) | 1.04 (0.72, 1.51) | 0.82 |
| Age (10 year increase) | 0.97 (0.70, 1.35) | 0.87 |
| Length of time from RP to SRT initiation (doubling) | 0.96 (0.78, 1.18) | 0.69 |
| Pathological tumor stage | Overall test of difference: P=0.003 | |
| T2 | 1.00 (reference) | N/A |
| T3a | 0.84 (0.44, 1.60) | 0.60 |
| T3b | 1.86 (0.96, 3.61) | 0.065 |
| Surgical margin |  |  |
| Negative | 1.00 (reference) | N/A |
| Positive | 1.11 (0.72, 1.69) | 0.64 |
| Gleason score | Overall test of difference: P=0.042 | |
| 3-6 | 1.00 (reference) | N/A |
| 7 | 1.10 (0.68, 1.78) | 0.70 |
| 8-10 | 1.99 (1.16, 3.41) | 0.012 |
| Pre-SRT hormone therapy |  |  |
| No | 1.00 (reference) | N/A |
| Yes | 1.31 (0.78, 2.19) | 0.31 |

RRs, 95% CIs, and p-values result from unadjusted Cox proportional hazards regression models. RP=radical prostatectomy; PSA=prostate-specific antigen; SRT=salvage radiation therapy. RR=relative risk; CI=confidence interval.
