# Supplementary material for: An Examination of the Association between FOXA1 Staining Level and Biochemical Recurrence following Salvage Radiation Therapy for Recurrent Prostate Cancer
Source: PLoS One. 2016 Mar 17;11(3):e0151785. doi: 10.1371/journal.pone.0151785 (PMC4795739; doi:10.1371/journal.pone.0151785)
Supplement: S2 Table — (DOC) [file pone.0151785.s003.doc]

**S2 Table: Association between FOXA1 staining level and biochemical recurrence following salvage radiation therapy separately for patients in the first and second staining batches**

|  | First staining batch | | Second staining batch | |
| --- | --- | --- | --- | --- |
| FOXA1 staining measure & group | RR (95% CI) | P-value | RR (95% CI) | P-value |
| FOXA1 H-score |  |  |  |  |
| Ordinal variable | 1.08 (0.96, 1.21) | 0.22 | 1.01 (0.85, 1.20) | 0.90 |
|  |  |  |  |  |
| ≤2 | 1.00 (reference) | N/A | 1.00 (reference) | N/A |
| >2 | 1.05 (0.56, 1.95) | 0.88 | 0.82 (0.46, 1.48) | 0.51 |
|  |  |  |  |  |
| ≤3 | 1.00 (reference) | N/A | 1.00 (reference) | N/A |
| >3 | 0.93 (0.51, 1.69) | 0.82 | 0.80 (0.42, 1.53) | 0.50 |
|  |  |  |  |  |
| ≤4 | 1.00 (reference) | N/A | 1.00 (reference) | N/A |
| >4 | 1.60 (0.86, 2.98) | 0.14 | 1.71 (0.67, 4.36) | 0.26 |
|  |  |  |  |  |
| FOXA1 staining percentage |  |  |  |  |
| Ordinal variable | 1.11 (0.87, 1.40) | 0.41 | 0.94 (0.74, 1.20) | 0.62 |
|  |  |  |  |  |
| ≤25% | 1.00 (reference) | N/A | 1.00 (reference) | N/A |
| >25% | 1.14 (0.56, 2.31) | 0.71 | 1.08 (0.57, 2.07) | 0.81 |
|  |  |  |  |  |
| ≤50% | 1.00 (reference) | N/A | 1.00 (reference) | N/A |
| >50% | 1.30 (0.71, 2.40) | 0.40 | 0.82 (0.46, 1.48) | 0.51 |
|  |  |  |  |  |
| ≤75% | 1.00 (reference) | N/A | 1.00 (reference) | N/A |
| >75% | 1.31 (0.73, 2.36) | 0.37 | 0.74 (0.38, 1.47) | 0.39 |
|  |  |  |  |  |
| FOXA1 staining intensity |  |  |  |  |
| Negative or weak | 1.00 (reference) | N/A | 1.00 (reference) | N/A |
| Moderate | 0.98 (0.53, 1.80) | 0.94 | 1.12 (0.50, 2.50) | 0.79 |

RRs, 95% CIs, and p-values result from unadjusted Cox proportional hazards regression models. Three separate dichotomizations of H-score were examined in Cox regression analysis that were defined based on the 25^th^ percentile (H-score=2), the 50^th^ percentile (H-score=3), and the 75^th^ percentile (H-score=4). FOXA1 staining percentage was scored as one of four categories (0% - 25%, 26% - 50%, 51% - 75%, 76% - 100%) and therefore the three possible dichotomizations of this variable were examined in Cox regression analysis. Given that there were only 6 patients with negative FOXA1 staining intensity, we combined this group with the 101 patients who had weak FOXA1 staining intensity for comparison with patients who had moderate FOXA1 intensity staining in Cox regression analysis. BCR=biochemical recurrence; RR=relative risk; CI=confidence interval.
